# Sequencing analysis reveals evidence of immune activation in advanced HER2 negative breast cancer responders treated with entinostat + nivolumab + ipilimumab

**DOI:** 10.21203/rs.3.rs-6580687/v1

**Published:** 2025-06-09

**Authors:** Evanthia Roussos Torres, Ludmila Danilova, Jospeh Tandurella, Edgar Gonzalez, Aaron Baugh, Batul Al-Zubeidy, Alexander Hopkins, Judy Murray, Melinda Downs, Luciane Kagohara, Elana Fertig, Roisin Connolly, Jesse Kreger, Vered Stearns

**Affiliations:** USC / Norris Comprehensive Cancer Center; Johns Hopkins; Johns Hopkins; Norris Comprehensive Cancer Center, Keck School of Medicine of the University of Southern California; Norris Comprehensive Cancer Center, Keck School of Medicine of the University of Southern California; USC / Norris Comprehensive Cancer Center; Dexter Biometry; Johns Hopkins School of Medicine; Johns Hopkins School of Medicine; Johns Hopkins University School of Medicine; University of Maryland; Breast Cancer Program, Sidney Kimmel Comprehensive Cancer Center at Johns Hopkins University; Department of Quantitative and Computational Biology, University of Southern California; Department of Medicine, Weill Cornell Medical College, Meyer Cancer Center

## Abstract

We analyzed the pathways associated with therapeutic response in 19 patients (5 responders) enrolled in a Phase Ib trial (NCT02453620) diagnosed with advanced HER2-negative breast cancer and treated with the HDAC inhibitor entinostat, in combination with dual immune checkpoint inhibitors (ICIs), nivolumab and ipilimumab. The analysis included gene expression and T-cell receptor (TCR) repertoire data derived from bulk RNA-seq and neoantigen data from whole exome sequencing. A total of 37 tumor biopsies were taken from six different metastatic sites at three timepoints: before treatment (baseline), after a two-week entinostat run-in (C1D1), and after the dual ICIs were added (week 8). Paired differential gene expression (DE) analysis between different timepoints revealed changes in immune-related pathways, such as interferon gamma and inflammatory response, IL6/JAK/STAT3 and IL2/STAT5 signaling, and allograft rejection after week 8 of triplet treatment. The DE analysis of response at each timepoint also revealed significant changes at baseline in immune-related pathways, such as inflammatory and interferon alpha and gamma responses, suggesting that responders may have had a preexisting immune tumor microenvironment (TME) that primed them for triplet therapy. Further analysis of the TME revealed significantly higher expression of genes representative of subsets of CD8+ T and plasma cells after entinostat treatment, indicating potential mechanisms of TME sensitization by HDACi. Genes representative of M1-like macrophages, pDC, memory CD4+ T, B, NK, lymphatic endothelial, Th1 cells, and memory B cells were significantly altered in responders after triplet treatment. PAM50 molecular subtype analysis revealed that basal and luminal B subtypes correlate with response. TCR analysis demonstrated higher diversity, and exploratory analysis of strong binding neoantigens showed fewer strong neoantigen binders in responders at week 8. In summary, the sequencing analysis reveals important changes in the immune landscape of responders to the combined treatment of entinostat and ICIs and suggests that pretreatment with entinostat may sensitize the immune TME to promote response.

Breast cancer remains a leading cause of cancer-related mortality among women worldwide, with diverse subtypes presenting unique challenges in treatment and prognosis^1^. Among these, triple-negative breast cancer (TNBC) and hormone receptor-positive (HR+) breast cancer represent two distinct clinical entities with significant implications for patient outcomes^2,3^. TNBC, characterized by the absence of estrogen receptor (ER), progesterone receptor (PR), and HER2 expression, accounts for approximately 15% of all breast cancers^4–10^. Treatment options involving checkpoint inhibition for advanced-stage TNBC is often limited to 1st line therapy only in PD-L1 + patients^11^. Entinostat, a histone deacetylase inhibitor (HDACi), has shown promise in combination with dual checkpoint inhibition. Studies have demonstrated that entinostat can enhance the efficacy of checkpoint inhibitors(ICI) like nivolumab, a PD-1 inhibitor, and ipilimumab, a CTLA-4 inhibitor, by modifying the tumor microenvironment to make it more responsive to treatment^12^. This combination therapy has resulted in notable response rates, particularly in patients with TNBC, who have limited treatment options^13^.

The NCT02453620 clinical trial was a Phase I study investigating the combination of entinostat, nivolumab, and ipilimumab in treating patients with advanced solid tumors^12^. The expansion cohort of the Phase Ib trial focused on evaluating the safety and preliminary efficacy of the triplet treatment in patients with advanced HER2-negative breast cancer. This Phase included 24 women, with 50% having hormone receptor-positive breast cancer and the other 50% having advanced triple-negative breast cancer. The primary endpoint was safety, and the results showed no dose-limiting toxicities. Secondary endpoints included overall response rate, clinical benefit rate, and progression-free survival. The overall response rate was 25%, with a higher response in triple-negative breast cancer (40%) compared to hormone receptor-positive breast cancer (10%). The clinical benefit rate was 40%, and progression-free survival at 6 months was 50%. These findings suggest that the combination therapy is safe and has potential efficacy, warranting further investigation in a Phase II trial.

Prior studies have also demonstrated that entinostat can decrease immune suppression and promote antitumor responses by altering the tumor microenvironment (TME). For instance, our study published in *Cancer Immunology Research* showed that entinostat treatment led to changes in multiple myeloid cell types, reducing immunosuppression and increasing antitumor immune responses in HER2 + breast tumors^14^. This study highlighted the reprogramming of tumor-associated macrophages from a protumor M2-like phenotype to an antitumor M1-like phenotype, which contributed to a more sensitized TME.

Here, we explored bulk sequencing data and its derivatives, such as gene expression, TCR repertoire, and neoantigen data from advanced TNBC and HR + breast cancer patients from the previously published Phase Ib of NCT02453620 trial^13^. We hypothesized that treatment with entionstat + nivolumab + ipilimumab would induce transcriptional changes in immune cells within the TME in support of an anti-tumor response .

## Results

### Study characteristics

**Figure 1:**
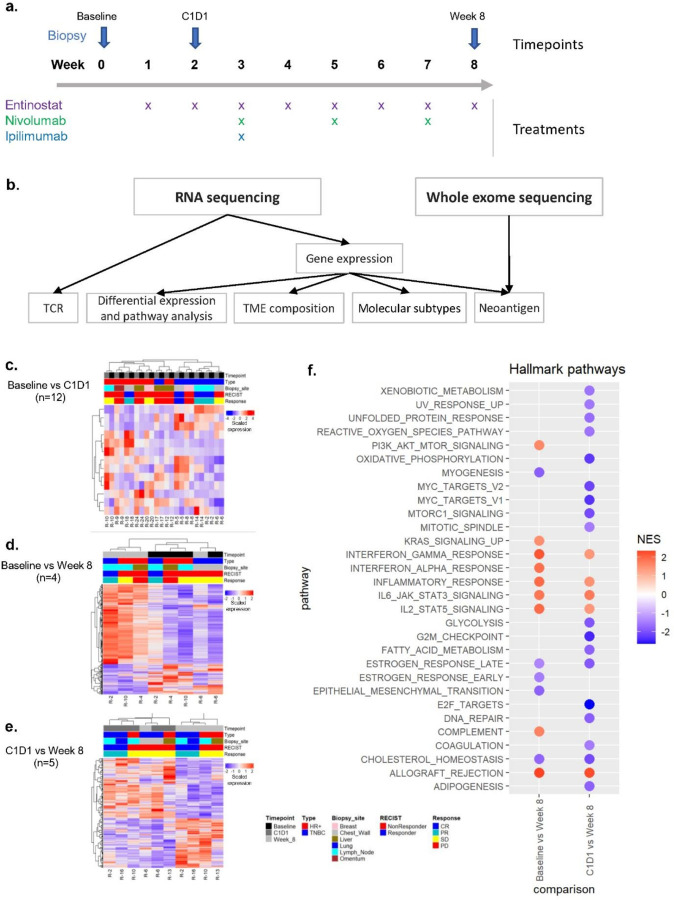
Study design and results of differential gene expression analysis of paired sample by treatment (timepoint). **a.** The study design. Tumor biopsies (blue arrow) were collected over three timepoints: Baseline, C1D1 (after 2-week entinostat Run-in), week 8 (after 6 weeks of combination therapy with entinostat + nivolumab + ipilimumab). **b.** The analysis chart that shows what analyses were performed using RNA and whole exome sequencing data. **c-e.**Heatmaps of significantly differentially expressed genes in analysis of paired samples between baseline and C1D1 (n=12 paired patient samples), baseline and week 8 (n=4 paired patient samples), and C1D1 and week8 (n=5 paired patient samples). The rows and the columns are clustered hierarchically with Pearson’s distance. **f.** Plot of significantly differentially enriched HALLMARK pathways (FDR-adjusted *p*-values < 0.05) for pair-wise comparisons between time points. Negative NES scores (blue) indicate pathways that are downregulated, while positive NES score (red) indicates pathway that are upregulated in week 8. NES, normalized enrichment score; TCR, T cell receptors; TME, tumor microenvironment

The bulk sequencing data were generated from tumor samples of patients from Phase Ib of the clinical trial NCT02453620^12^. In total, there were 37 tumor biopsy samples collected from 19 patients (five responders and 14 non-responders) with the evaluable disease across six tissue sites and three timepoints: before treatment (baseline), after a two-week entinostat run-in (C1D1), and after dual ICIs were added in (week 8) (Fig. 1a). Bulk RNA sequencing reads were used to generate gene expression and TCR repertoire data, while bulk whole exome sequencing data were used to generate neoantigen binding affinity (Fig. 1b). In turn, gene expression data were entered into differential expression (DE), pathway, and tumor microenvironment (TME) analyses, as well as molecular subtype classifications. Additionally, we used gene expression data to inform the selection of strong binding neoantigens (see details in Methods). The number of samples varied across timepoints for various reasons such as missed biopsy appointments, insufficient biopsy material, or insufficient quality to perform sequencing (Supplementary Table 1).

The observed site-specific effect on RNA-seq results has the potential to bias differential expression analysis due to the heterogeneity of tissue biopsy sites. Most biopsies were collected from the liver (n = 10) and lymph nodes (n = 10), followed by eight chest wall, four breast, three lung, and two omental samples (Supplementary Table 2). In clinical trials investigating the efficacy of ICIs in breast cancer, patients with certain sites of metastasis were less likely to respond to ICIs^15–18^. Liver metastases have been characterized as more immunosuppressed, and lung metastases as more immunoresponsive^19,20^. To study varied responses at measurable metastatic sites within each patient, we reviewed RECIST imaging reports. The review illustrated that out of five responders, three had metastases to the lungs, one had metastasis in the breast, chest, or skin, and all demonstrated metastases to the lymph nodes (Supplementary Table 3). All these lesions were either stable or shrank. Of note, none of the responders had liver metastases, whereas seven out of 14 non-responding patients had liver metastases that all demonstrated notable growth. Other sites of metastasis in non-responders included four in the lung, three in the breast and peritoneal/retroperitoneal, two in the lymph nodes and chest, and one in the axillary, skin, adrenal, pleural, and other (Supplementary Table 3). To address the diversity of site-specific responses, we included tissue as a covariate in the DE analysis of response.

Another challenge was the small number of RECIST responders (n = 5), which was even smaller for baseline (n = 4) and week 8 (n = 3) timepoints (Supplementary Table 1). This small number of patients is typical for Phase I trials, which primarily focus on assessing the safety, side effects, and optimal dosing of new treatments. As a result, these trials usually include a limited number of participants who have not responded to other treatments.

### Gene expression changes by treatment

To study gene expression changes with treatments, we performed DE analysis using paired samples between timepoints (Fig. 1b–e, EDFig. 1, Supplementary Table 4–8). First, to study the effect of entinostat treatment, we compared paired samples between baseline and C1D1 from 12 patients. This analysis yielded 13 significant genes (one gene upregulated at baseline and 12 genes upregulated at C1D1), and changes in gene expression were mostly due to patient heterogeneity rather than due to entinostat treatment as shown on the heatmap in Fig. 1b (EDFig. 1a, Supplementary Table 4). A similar lack of major DE transcriptional differences after entinostat treatment was observed in the Phase II clinical trial in metastatic pancreatic ductal adenocarcinoma^21^. Gene set enrichment analysis of hallmark pathways using results from this DE analysis did not yield any significant pathways.

Next, to study the effect of the combined treatment of entinostat, nivolumab, and ipilimumab compared to baseline, we used paired samples between baseline and week 8 from four patients. This analysis yielded 495 significantly DE genes (133 genes upregulated at baseline and 362 genes upregulated at week 8) and 13 significantly enriched hallmark pathways (Fig. 1c, EDFig. 1b, Supplementary Table 5, 6). Analysis of paired samples between C1D1 and week 8 from five patients yielded 231 significantly DE genes (127 genes upregulated at C1D1 and 104 genes upregulated at week 8) and 23 significantly enriched hallmark pathways (Fig. 1d, EDFig. 1c, Supplementary Table 7, 8). Seven common hallmark pathways, including four immune-related pathways (interferon gamma and inflammatory response, IL6/JAK/STAT3 and IL2/STAT5 signaling, and allograft rejection), were upregulated at week 8 after ICI, and the only downregulated pathway was the cholesterol homeostasis pathway (Fig. 1e).

In summary, there were significant changes in mRNA levels in samples after the combination of entinostat and ICI, especially in immune-related pathways, but there were not many significant changes in mRNA levels in samples after two weeks of entinostat treatment alone.

### Gene expression changes by response to treatment

**Figure 2:**
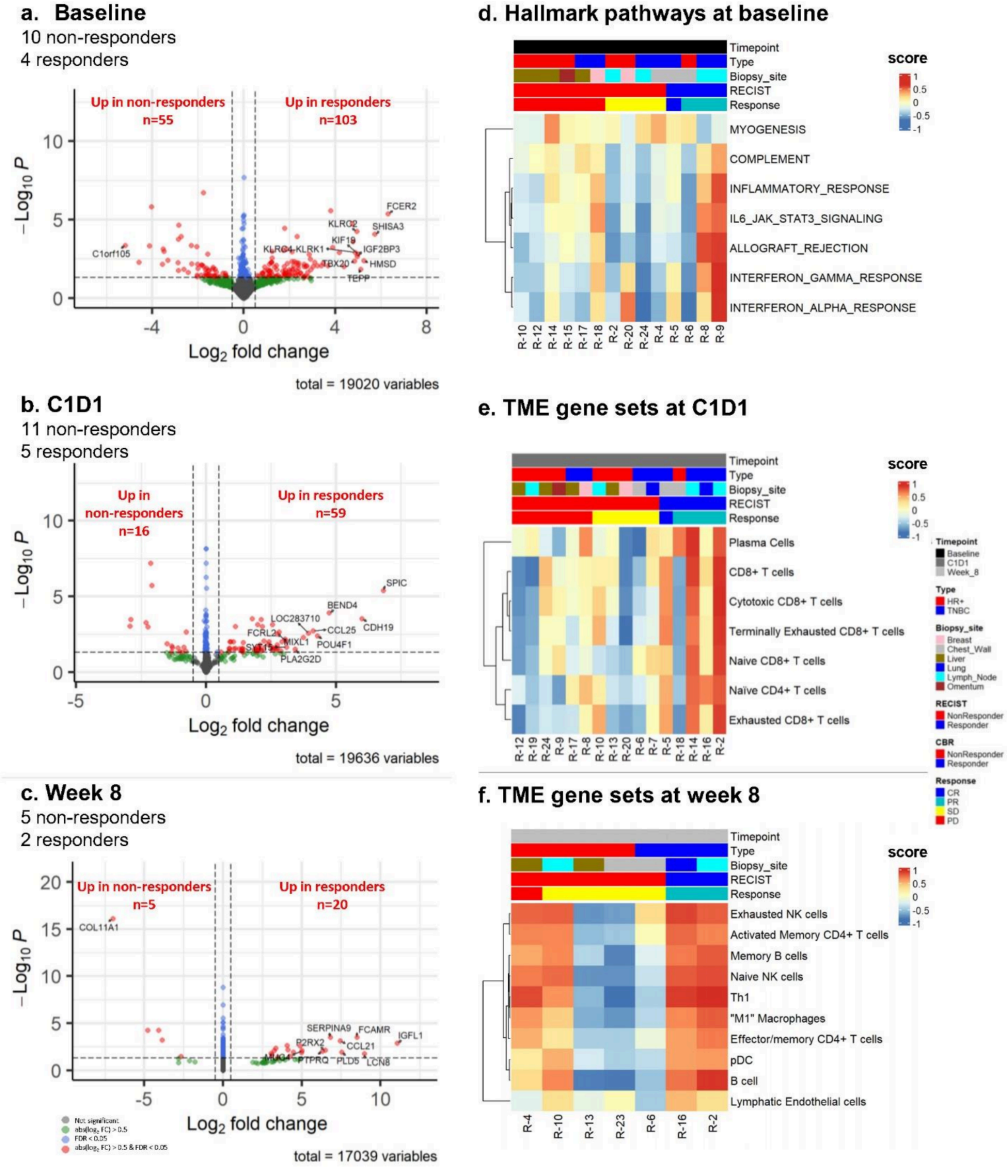
Results of differential gene expression and gene set variation analyses of responders vs non-responders at each timepoint. **a-c.** Volcano plots of the results of differential gene expression analysis between responders and non-responders at baseline (a), C1D1 (b), and week 8 (c). The analysis was performed using the DESeq2 package. Genes with the absolute log2-fold changes > 0.5 and FDR > 0.05 are in green, genes with the absolute log2-fold changes < 0.5 and FDR < 0.05 are in blue, and genes the absolute log2-fold changes > 0.5 and FDR < 0.05 are in red. **d.** A heatmap of gene set scores of HALLMARK pathways that were significantly highly enriched (FDR-adjusted *p*-values < 0.05) in responders and non-responders at baseline. Gene set enrichment analysis was performed by the fgsea package, and gene set scores were calculated by the GSVA package. The columns are ordered by RECIST response, and the rows are clustered hierarchically with Pearson’s distance. **e-f.**Heatmaps of gene set scores of TME gene sets with two-sided Wilcoxon sum rank test p-values < 0.1 between responders and non-responders at C1D1 and week 8. The columns are ordered by RECIST response, and the rows are clustered hierarchically with Pearson’s distance. TME, tumor microenvironment

To see if there were differences in gene expression associated with response to treatments, we performed DE analysis at each timepoint comparing RECIST responders vs. non-responders (Fig. 2, EDFig. 2, Supplementary Tables 9–13). The trial reported five RECIST responders, including patients with complete or partial response (CR or PR), and 15 non-responders, including patients with stable or progressive disease (SD or PD)^13^. The RNA sequencing data was available for different subsets of patients at different timepoints. The analysis of 10 non-responders and four responders at baseline yielded 158 significantly DE genes (55 downregulated and 103 upregulated genes in responders) (Fig. 2a, EDFig. 2a, Supplementary Table 9). We did the literature search to see how many immune-related genes were among the top ten significantly DE genes. Among the top ten significantly up- or down-regulated genes, 50% and 70% were immune-related, respectively, with a high representation of T cell/NK cell marker genes upregulated in responders, such as *KLRK1*, *KLRC2*, and *KLRC4* (Supplementary Table 10). Furthermore, the most upregulated gene in responders, *FCER2A*, is associated with tumor suppressor function and is mostly expressed in B cells. These results suggest that B cells and T cells/NK cells were present in higher numbers in the tumors of responding patients at baseline.^22^ Additionally, seven hallmark pathways were significantly enriched – six immune-related pathways were highly enriched in responders, and only the myogenesis pathway was enriched in non-responders (Fig. 2d, Supplementary Table 11).

The DE analysis between responders and non-responders at C1D1 yielded 75 significantly DE genes (16 downregulated and 59 upregulated genes in responders), but no significantly enriched hallmark pathways (Fig. 2b, EDFig. 2b, Supplementary Table 12). The literature search showed that among the top ten significantly up- or down-regulated genes, 40% and 50% were immune-related, respectively (Supplementary Table 10). Of note, one of the top downregulated genes in responders was *TREM2*, which is associated with immunosuppressive macrophages in tumors.

And the same DE analysis at week 8 resulted in only 25 significantly DE genes (5 downregulated and 20 upregulated genes in responders), and no significantly enriched pathways (Fig. 2c, EDFig. 2c, Supplementary Table 13). The literature search showed that among the top ten significantly up- or down-regulated genes, 80% were immune-related (Supplementary Table 10).

We also performed similar DE analyses using clinical benefit rate (CBR), where CBR responders included RECIST responders and patients with stable disease, and non-responders were patients with progressive disease. The analyses were only feasible at baseline and C1D1 because there was only one CBR non-responder at week 8, and these analyses yielded very few expressional changes (EDFig. 3, Supplementary Tables 14, 15).

Overall, these findings highlight the significant role of immune-related gene expression in treatment response, particularly at baseline, and underscore the complexity of gene expression dynamics over the course of treatment.

### Tumor microenvironment analysis

To study changes in the tumor microenvironment (TME) due to treatment, we ran CIBERSORTx analysis^23^ to estimate the proportions of 22 immune cell populations (EDFig. 4a, Supplementary Table 16). We found that proportions of monocytes were higher in non-responders at baseline (p-value = 0.008, EDFig. 4b, Supplementary Table 17), but entinostat induced a bigger change in proportion of monocytes specifically in responders (p = 0.028) (EDFig. 4c). To more closely study the TME, we used manually curated markers of 61 gene sets representing cell types in the microenvironment (Supplementary Table 18). Using these markers and gene expression data, we derived per sample gene set scores using gene set variation analysis implemented in the GSVA R package^24^ (Supplementary Table 19). To derive scores, we used only markers that are expressed in a cell type. Subsequently, we performed the pairwise comparison of these scores between responders and non-responders at each timepoint. CD8 + T and plasma cell scores were significantly higher after entinostat treatment (Fig. 2e, Supplementary Table 20) while M1-like macrophages, pDC, Memory CD4 + T, B, NK, Lymphatic Endothelial, Th1 cells, and memory B cells were higher after the triplet treatment (Fig. 2d, Supplementary Table 20). These findings suggest that the expression of genes representative of immune cells in the TME had higher expression in responders at both C1D1 and week 8. Overall, these results indicated that treatment-induced changes in the tumor microenvironment, particularly in immune cell populations, are more pronounced in responders, highlighting the dynamic interplay between treatment and immune response.

### Molecular subtypes and response

**Figure 3:**
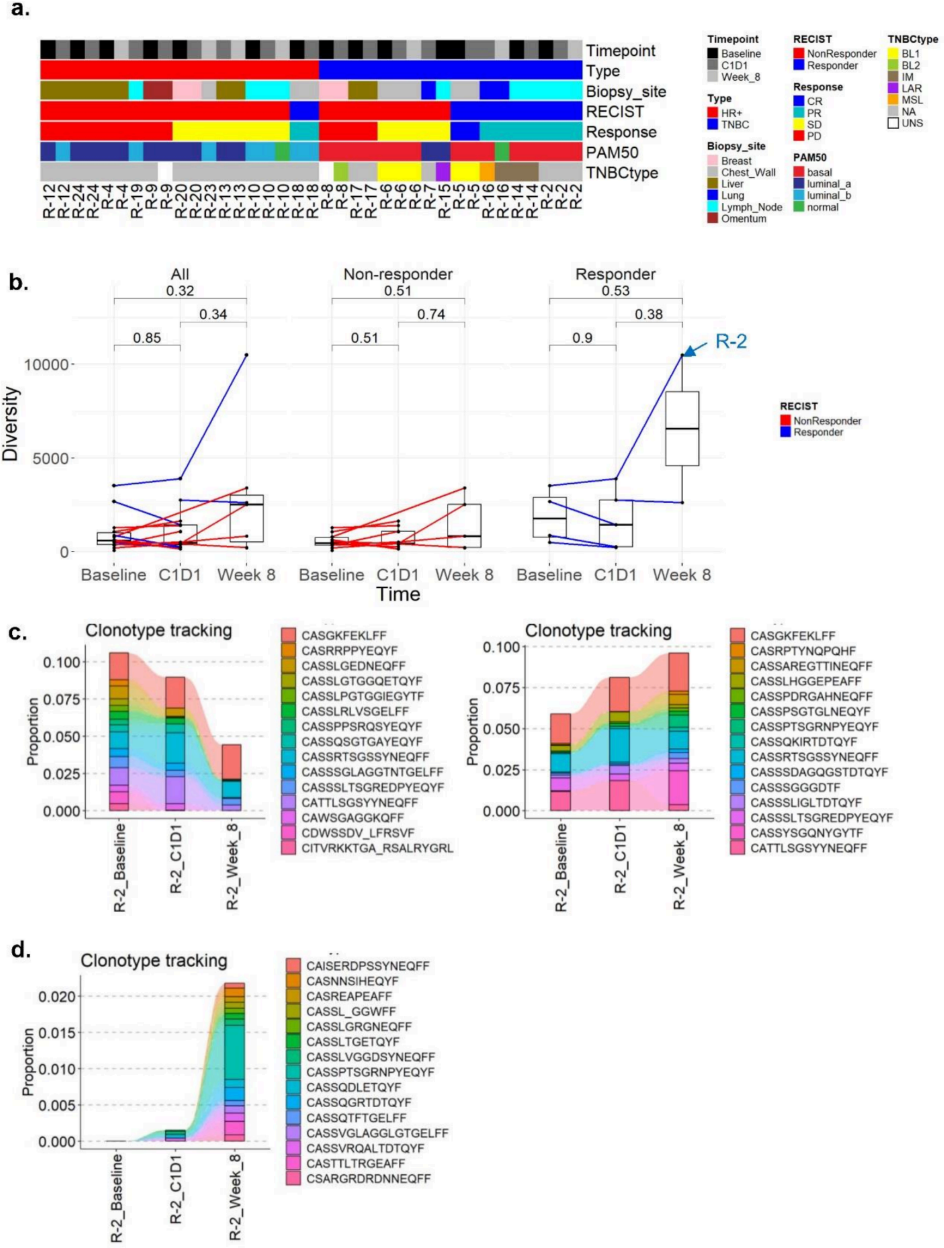
Molecular subtypes and results of T cell receptor repertoire analysis. **a.** The PAM50 and TNBCtype molecular subtype prediction results along with clinical information and response for all samples (n=37). **b.** Boxplots of the diversity of TCR repertoires in all samples over timepoints, as well as for non-responders and responders. The two-sided Wilcoxon sum rank test p-values are shown. **c.** Clonotype tracking plots of the top 15 of the most abundant clones in baseline (left panel) and in week 8 (right panel) in responder R-2. **d.** Clonotype tracking plot of the 15 of the most abundant clones at week 8 that are not present at baseline in responder R-2. TNBCtypes: BL1 - basal-like 1, BL2 - basal-like 2, IM – immunomodulatory, MSL - mesenchymal stem-like (MSL), LAR - luminal androgen receptor, UNS – unstable, NA – not available.

Given the heterogeneity among patients and within pathologic breast cancer subtypes, we used multiple molecular subtyping tools to help further characterize our patient cohort and identify potential trends in responders vs. non-responders. We first used the PAM50 classifier, which assigned samples into five subtypes (luminal A, luminal B, HER2-enriched, basal-like, and normal-like) based on the expression profile of 50 genes^25^. We found that the PAM50 subtypes were significantly associated with breast cancer subtypes (Chi-squared test p-value 0.002), since most of the HR + samples were classified as luminal A and B subtypes, and most of the TNBC samples were classified as basal-like (Fig. 3a, Supplementary Table 21). None of the samples were classified as HER2 or normal-like at baseline consistent with reported histologic subtypes from the clinical trial^12,13^. The PAM50 subtypes were also significantly associated with response (Chi-squared test p-value 0.04). Four out of five responders, identified as TNBC, were classified as basal-like, and the one HR + responder was classified as luminal B, consistent with recently reported data showing luminal B breast cancers may be more responsive to checkpoint inhibition^26–28^.

To further classify molecular subtypes, we utilized TNBCtype, another in silico classification tool^29,30^ which classified tumors into six subtypes: two basal-like (BL1 and BL2), an immunomodulatory (IM), a mesenchymal (M), a mesenchymal stem-like (MSL), and a luminal androgen receptor (LAR). Applying TNBCtype to our cohort, we found that the TNBC patients were classified into the following subtypes: two as BL1, one as BL2, one as IM, one as MSL, and one as LAR subtype, but none as M (Fig. 3a). One TNBC non-responder (R-8) at baseline could not be stably classified to one subtype because of very similar coefficients for both IM (0.178) and BL2 (0.165) subtypes (Supplementary Table 22). Interestingly, the C1D1 sample from the HR + patient (R-9) was also unstably classified as BL2 (0.522), MSL (0.502), and LAR (0.453). Classification of TNBC responders into BL1, MSL, and IM subtypes is consistent with published results using this tool that has reported the correlation of these subtypes with the level of tumor-infiltrating lymphocytes and potential for response to immunotherapy^31^. The TNBCtype BL2, BL1, and LAR subtypes were identified in non-responders, which was inconsistent with predictions from previously reported TNBCtype^32^. Of note, not all patients had baseline samples available, and as such, these subtype classification may not be as relevant in tumors after treatment, Nonetheless, data showed a change in TNBCtype and PAM50 subtypes after treatment, a measure that is not typically reported, but which could inform how treatment has affected tumor cell phenotype. For example, in responder R-16 the TNBCtype subtype changed after treatment from MSL at C1D1 to IM at week 8, and this same patient demonstrated a change in the PAM50 subtype from basal to normal. These changes may represent what is left in the tumor bed. In addition, TNBC non-responder (R-8) changed TNBCtype subtype from unstable at baseline to BL2 after entinostat treatment, which could have treatment implications (i.e. make the tumor less responsive to treatment). Classification of molecular subtypes could be used to guide future investigations.

### T cell receptor repertoire and neoantigen analyses

The higher gene set scores of T cells in responders after treatment led us to study T cell receptor (TCR) repertoires. These receptor sequences were successfully extracted from the unaligned reads in the RNA-seq data using MiXCR and then compared between treatments and responders. The clonal diversity of TCR repertoires showed a trend towards higher numbers after triplet treatment (Fig. 3b, Supplementary Table 23). Responder R-2 had the highest number of unique clonotypes and diversity among all patients and timepoints, which was not unexpected, given that the samples obtained from R-2 patient were from lymph node tissue. We observed that expanded TCR clonotypes detected in the baseline sample of that responder remained through the course of treatment (Fig. 3c). Moreover, we were able to detect 9435 (79% of the repertoire) out of 10200 clonotypes that were not present at baseline, but showed expansion in response to the therapy (Fig. 3d).

We also utilized neoantigen binding affinity generated from whole exome sequencing to study changes in the number of strong binders over the treatment course and between responders and non-responders. The somatic mutations and tumor mutational burden for these patients were previously published^13^. Here, we performed the exploratory analysis that demonstrated a lower number of strong binders in responders at week 8 (EDFig. 5, Supplementary Table 23).

Overall, these analyses revealed that treatment enhances T cell receptor diversity and neoantigen response in responders, suggesting a robust adaptive immune response to therapy.

## Discussion

The study involved bulk sequencing of 37 tumor biopsy samples from 19 patients across six tissue sites and three timepoints (baseline, C1D1, and week 8). Despite challenges posed by the diversity of tissue biopsy sites and the small number of responders, significant findings emerged. Differential expression (DE) analysis between baseline and C1D1 identified 13 significant genes, with changes primarily due to patient variation rather than entinostat treatment. No significant pathways were identified. However, combined treatment with entinostat, nivolumab, and ipilimumab showed more pronounced effects, with DE analysis between baseline and week 8 revealing 495 significant genes and 13 enriched hallmark pathways. Analysis between C1D1 and week 8 identified 231 significant genes and 23 enriched pathways, with five pathways in common, including four immune-related pathways, upregulated at week 8 after ICI treatment. Gene expression analysis comparing RECIST responders and non-responders at each timepoint revealed significant differences, particularly at baseline, where 158 significant genes and seven enriched pathways were identified, with immune-related pathways enriched in responders. Tumor microenvironment (TME) analysis showed higher scores in responders for CD8 + T and plasma cells after entinostat treatment, and for various other immune cell population after triplet treatment. T cell receptor (TCR) repertoire analysis indicated higher clonal diversity after triplet treatment, with patient R-2 showing the highest diversity. Neoantigen analysis showed a trend of fewer strong binders in responders at week 8. Finally, PAM50 subtype analysis revealed that HR + samples were enriched with luminal A and B subtypes, while TNBC samples were predominantly basal.

Our study provides insights into the gene expression changes associated with entinostat treatment and its combination with dual immune checkpoint inhibitors (ICIs) in tumor samples from patients. The differential expression (DE) analysis between baseline and C1D1 revealed minimal significant changes, suggesting that the short-term entinostat treatment alone may not induce substantial alterations in gene expression. This finding aligns with previous studies, such as the Phase II clinical trial in metastatic pancreatic ductal adenocarcinoma, which also reported a lack of significant DE after two weeks of entinostat treatment^21^. The limitations of the two-week run in are that changes may be initiated that influence remodeling of the TME but are not measurable at that short time point. Additional studies would be necessary to determine if a run in is necessary to achieve a response or if concurrent treatment with entinostat and dual ICI would be sufficient.

In contrast, the combined treatment of entinostat, nivolumab, and ipilimumab resulted in more pronounced gene expression changes. The DE analysis between baseline and week 8, as well as between C1D1 and week 8, identified numerous significantly differentially expressed genes and enriched hallmark pathways, particularly those related to immune responses. The upregulation of immune-related pathways, such as interferon-alpha/gamma response, inflammatory response, IL6/JAK/STAT3 signaling, and allograft rejection, indicates a robust activation of the immune system following the addition of ICIs. Furthermore, most of these pathways are associated with an anti-tumor response, although the interferon-gamma and inflammatory response can have either pro- or anti-tumoral effects depending on the context, which can account for the lack of response in patients in which these changes were observed^33–35^. This suggests that the combination therapy may enhance anti-tumor immunity, potentially contributing to improved clinical outcomes. The molecular subtype analysis using the PAM50 provided additional context for the observed gene expression changes in tumor cells, with HR + samples enriched for Luminal A and B subtypes and TNBC samples being predominantly basal. The TNBCType analysis also highlights the additional heterogeneity of tumor samples and the potential for molecular subtyping to assist in response predictions. This subtype distribution may influence the differential response to treatment and warrants further investigation, as demonstrated in ongoing clinical trials.

The comparison of gene expression between RECIST responders and non-responders further supports the role of immune activation in treatment response. At baseline, responders exhibited significant enrichment of immune-related pathways and genes associated with the presence of anti-tumoral cell types including NK cells, T cells, and B cells, highlighting the importance of pre-existing immune activity in predicting response to therapy. The tumor microenvironment (TME) analysis corroborated these findings, showing higher scores for immune cell markers in responders, both after entinostat treatment and triplet therapy. This underscores the critical role of the TME in mediating treatment efficacy. Among the significantly downregulated genes in responders at C1D1 that is maintained at week 8, the Triggering Receptor Expressed on Myeloid cells 2 (TREM2) gene, which is expressed on macrophages, dendritic cells, and microglia, plays a significant role in immune responses and inflammation^36^. TREM2 has been associated with immunosuppressive functions in macrophages, where it can inhibit cytokine production and macrophage activation^37^. This immunosuppressive role is crucial in creating an immunosuppressive microenvironment that can promote tumor growth^38^. Additionally, TREM2 has been linked to resistance to immune checkpoint inhibitors (ICIs)^39^ and notably was recently observed to also be induced during neoadjuvant ICI treatment of pancreatic tumors^40^. TREM2 can negatively regulate antitumor immunity by inhibiting T cell proliferation and other immune responses, making TREM2 a potential target for improving the efficacy of cancer immunotherapies.

While our analysis uncovered immunosuppressive macrophages, it also indicated a role for T cell response in ICI treatment. T cell receptor (TCR) repertoire analysis revealed increased clonal diversity following triplet treatment, particularly in patient R-2, suggesting that the combination therapy may promote a more diverse and potentially more effective T cell response. Additionally, our study’s observation of increased clonal diversity in T cell receptor (TCR) repertoires after triplet treatment aligns with earlier reports suggesting that entinostat can enhance the effectiveness of ICIs by promoting a more diverse and robust T cell response^41^. This increased diversity is indicative of a more effective antitumor-immune response, which has been associated with better clinical outcomes. Taken together with exploratory neoantigen analysis, which showed fewer strong binders in responders at week 8, these data suggest a more efficient immune clearance of cancer cells expressing neoantigens in responder patients, as observed by Truong *et al*. with entinostat treatment in preclinical models of bladder cancer^41^.

Finally, the consideration of clinical factors, such as sites of metastatic disease, suggests that there may be biologically meaningful patterns that inform response. The fact that all responders had lung metastases, while none of the patients with liver metastases responded, indicates that investigating differences in the immune response by the site of disease could elucidate immunologic mechanisms contributing to outcomes. Still, we note that these observations are limited by the sample size of our clinical trial. Future investigations could explore differential immune activation at varied sites of disease, and the consideration of disease burden location could be incorporated into future clinical trials. A further limitation of our study arises from potential biases in sample collection, such as missed biopsy appointments or insufficient biopsy material, which could affect the results. The exploratory nature of our neoantigen analysis, although backed by preclinical evidence, requires validation in larger cohorts. Additionally, the lack of functional validation of gene expression changes and pathways through experimental studies limits the biological relevance of our findings. Addressing these limitations in future research will help build on our current findings, advancing the field of cancer immunotherapy and ultimately improving patient care.

In summary, our results suggest that while short-term entinostat treatment alone may have a limited impact on gene expression, its combination with ICIs leads to significant immune activation and gene expression changes, particularly in responders. These findings highlight the potential of combination therapies to enhance anti-tumor immunity and improve clinical outcomes in cancer patients.

## Methods

### Statistical considerations and reproducibility

FDR adjustment for multiple test correction was performed for DE and gene set analyses. FDR-adjusted p-values less than 0.05 were considered significant. In all other analyses the multiple test adjustment was not considered because of the limited number of samples and the exploratory nature of the analyses.

### RNA and whole exome sequencing

RNA and whole exome sequencing were conducted at Personalis Inc. (Menlo Park, CA, USA). The ImmunoID NeXT analysis pipeline was used to generate gene expression counts, neoantigen binding prediction, and clonality of the TCR repertoire. DNA and RNA were dual-isolated from FFPE samples utilizing the AllPrep DNA/RNA FFPE Tissue kit (Qiagen). For DNA extraction of matched normals, peripheral blood mononuclear cells (PBMC) were submitted and the AllPrep DNA/RNA Mini Kit (Qiagen) was used for isolation. Exome capture was performed using Agilent SureSelect Clinical Research Exome v2 (SSCRv2) according to manufacturers’ recommendations. Additional supplementation with Personalis ACE proprietary target probes was performed to enhance coverage in difficult to sequence regions within sets of biomedically and medically relevant genes. Details regarding Personalis ACE assay design are described further in Patwardhan et al. 2015^42^. In brief, manufacturer protocols were modified to adjust the average library insert length to approximately 250 bp and the use of KAPA HiFi DNA Polymerase (Kapa Biosystems) for DNA sequencing and Stranded RNA Sequencing kit (Kapa Biosystems) for RNA Sequencing. Sequencing was performed on NovaSeq 6000 (Illumina, San Diego, CA, USA) sequencers with paired-end 2 × 150 bp read lengths and Illumina’ s proprietary reversible terminator-based method. DNA for the matched normal sample was sequenced at an average depth of coverage of ~ 150X. For the tumor specimens, DNA was sequenced to an average depth of coverage of ~ 300X across the 74.8 Mb NeXT assay genomic footprint. For RNA sequencing tumor specimens were sequenced to an average output of 100 million paired end reads (total of 200 M reads).

### Differential gene expression

We evaluated sequencing quality from the distribution of gene counts as visualized in a boxplot of log counts. We observed no samples with zero median expression, reflective of a low read count, so all samples had good quality. We used principal component analysis (PCA) of the variance stabilization transform (vst) RNA-seq data to evaluate sample clustering. We performed differential gene expression analysis using the DESeq2 package (v1.46.0)^43^. We performed DE analysis in two ways. First, we analyzed paired analysis between pairs of timepoints, and then we compare responders and non-responders within each time point including tissue of biopsy site as a covariate in model. For a paired analysis between timepoints, we used samples from 12 patients between baseline and C1D1, four patients between baseline and week 8, and five patients between C1D1 and week 8. We included the patient ID as a covariate in our model, along with timepoint. For a response analysis, the number of samples varied at each time point due to various reasons such as missed biopsy appointments, insufficient biopsy material, or insufficient quality to perform RNA sequencing, so out of five responders, we had four, five and two samples at baseline, C1D1, and week 8, respectively. The number of samples per time point and response can be found in Supplementary Table 1.

Genes were statistically significant if the absolute log2-fold changes after shrinkage were greater than 0.5 and the FDR-adjusted p-value was below 0.05 (Supplementary Tables 4, 5, 7, 9, 12–15). Gene set statistics were run with fgsea (v1.32.0)^44^ using msigdbr v7.5.0 pathways annotated in the HALLMARK database^45^. Gene sets were considered significantly enriched for FDR-adjusted p-values below 0.05 (Supplementary Tables 6, 8, 11). The results were visualized with ggplot2 (v3.5.1)^46^, EnhancedVolcano (v1.24.0), and ComplexHeatmap (v2.20.0)^47^.

### Gene set variation analysis

Using gene expression counts and gene sets (TME or HALLMARK), we derived gene set scores per sample with the help of gene set variation analysis implemented in the GSVA (v2.0.2) package. The TME gene set was compiled from various sources after conducting a literature search for cell-type specific markers, prioritizing research articles describing human single cell RNA-sequencing datasets. The references for each TME gene set can be found in Supplementary Table 18. TME gene set scores were compared between responders and non-responders at each timepoint using two-sided Wilcoxon rank-sum test. For visualization using ComplexHeatmap (v2.20.0)^47^, we used scores of gene sets that passed p-value threshold less than 0.1.

Using gene expression counts and cell type markers as gene sets, we derived gene set scores per sample and gene set with the help of gene set variation analysis implemented in the GSVA (v2.0.2) package (Supplementary Table 19).

### T cell receptor repertoire analysis

T cell receptor repertoires derived from RNA sequencing data were estimated using MiXCR and provided by Personalis Inc. (Menlo Park, CA, USA). We loaded the provided data into R programming environment using the immunarch package (v0.9.1) and calculated the diversity using the repDiversity function with the chaos1 method. The diversity was visualized as boxplots with pairwise two-sided Wilcoxon rank-sum test p-values. The trackClonotypes function was used to track 15 of the most abundant clonotypes in baseline and week 8 in patient R-2.

### Neoantigen analysis

Neoantigen binding affinity was estimated using NetMHCIIPan 4.0 by Personalis Inc. (Menlo Park, CA, USA). To calculate the number of strong binders per sample, we took only peptides of expressed genes, and then for every peptide, we took the minimal predicted HLA rank. After that, we calculated the number of peptides that have rank less than 2 as recommended by Reynisson et al^48^ to be a strong binder. The number of strong neoantigen binders was visualized as boxplots with pairwise two-sided Wilcoxon rank-sum test p-values.

### Molecular subtypes and cell type proportions

To assign PAM50 subtypes to our samples, we applied the pam50 package (v0.2.0)^25^ to the gene expression data (Supplementary Table 21). To classify samples according to TNBCtype subtypes, we uploaded the gene expression data to the TNBCtype webtool (https://cbc.app.vumc.org/tnbc/). Out of 37 samples, the tool was able to classify 13 samples from six TNBC patients and one week 8 sample from HR + patient R-9 (Supplementary Table 22).

### CIBERSORTx analysis

Using gene expression counts, we applied CIBERSORTx tool (https://cibersortx.stanford.edu/) ^23^ to estimate proportions of 22 immune cell populations (Supplementary Table 16). Then the estimates were uploaded into R/Bioconductor and paired pairwise two-sided Wilcoxon sum-rank test was applied to compare samples from the same patients between pairs of timepoints. Additionally, pairwise two-sided Wilcoxon sum-rank test was applied to the estimated proportions to compare responders and non-responders within each timepoint and to the change in proportions between pairs on timepoints to study the treatment effect difference between responders and non-responders (Supplementary Table 17). The cell proportions were visualized as a heatmap and boxplots with pairwise two-sided Wilcoxon rank-sum test p-values.

## Supplementary Material

Supplement 1

## Data Availability

All of the datasets generated during the current study that can be shared are available as Supplementary Tables. The raw sequencing data are not publicly available due to a lack of consent for data sharing. Source data are provided with this paper and are also available at https://github.com/OncologyQS/J15221_RNAseq_paper/tree/main/input_data.
