# Supplementary material for: Sequencing analysis reveals evidence of immune activation in advanced HER2 negative breast cancer responders treated with entinostat + nivolumab + ipilimumab
Source: Res Sq. 2025 Jun 9:rs.3.rs-6580687. Preprint. [Version 1] doi: 10.21203/rs.3.rs-6580687/v1 (PMC12204493; doi:10.21203/rs.3.rs-6580687/v1)
Supplement: Supplement 1 [file NIHPPrs6580687v1-supplement-1.pdf]

Supplementary Files

This is a list of supplementary files associated with this preprint. Click to download.

- Supplementarytable12.xlsx
- Supplementarytable3RECISTchanges.xlsx
- Supplementarytable48DEtreatment.xlsx
- Supplementarytable915DEresponse.xlsx
- Supplementarytable1620TME.xlsx
- Supplementarytable2122pam50TNBCtype.xlsx
- Supplementarytable23diversityneoantigen.xlsx
- ExtendedFigures.docx
